# Changes in Intestinal Glucocorticoid Sensitivity in Early Life Shape the Risk of Epithelial Barrier Defect in Maternal-Deprived Rats

**DOI:** 10.1371/journal.pone.0088382

**Published:** 2014-02-20

**Authors:** Nabila Moussaoui, Viorica Braniste, Afifa Ait-Belgnaoui, Mélissa Gabanou, Soraya Sekkal, Maiwenn Olier, Vassilia Théodorou, Pascal G. P. Martin, Eric Houdeau

**Affiliations:** 1 Intestinal Development, Xenobiotics & Immunotoxicology, Institut National de la Recherche Agronomique (INRA), Research Centre in Food Toxicology (Toxalim), Toulouse, France; 2 Neurogastroenterology & Nutrition, INRA, Toxalim, Toulouse, France; 3 Integrative Toxicology & Metabolism, INRA, Toxalim, Toulouse, France; 4 GeT-TRiX facility, INRA, Toxalim, Toulouse, France; Institut Pasteur de Lille, France

## Abstract

Glucocorticoids (GC) contribute to human intestine ontogeny and accelerate gut barrier development in preparation to birth. Rat gut is immature at birth, and high intestinal GC sensitivity during the first two weeks of life resembles that of premature infants. This makes suckling rats a model to investigate postpartum impact of maternal separation (MS)-associated GC release in preterm babies, and whether GC sensitivity may shape MS effects in immature gut. A 4 hours-MS applied once at postnatal day (PND)10 enhanced plasma corticosterone in male and female pups, increased by two times the total *in vivo* intestinal permeability (IP) to oral FITC-Dextran 4 kDa (FD4) immediately after the end of MS, and induced bacterial translocation (BT) to liver and spleen. Ussing chamber experiments demonstrated a 2-fold increase of permeability to FD4 in the colon immediately after the end of MS, but not in the ileum. Colonic permeability was not only increased for FD4 but also to intact horseradish peroxidase 44 kDa in MS pups. *In vivo*, the glucocorticoid receptor (GR) antagonist RU486 or ML7 blockade of myosin light chain kinase controlling epithelial cytoskeleton contraction prevented MS-induced IP increase to oral FD4 and BT. In addition, the GR agonist dexamethasone dose-dependently mimicked MS-increase of IP to oral FD4. In contrast, MS effects on IP to oral FD4 and BT were absent at PND20, a model for full-term infant, characterized by a marked drop of IP to FD4 in response to dexamethasone, and decreased GR expression in the colon only compared to PND10 pups. These results show that high intestinal GC responsiveness in a rat model of prematurity defines a vulnerable window for a post-delivery MS, evoking immediate disruption of epithelial integrity in the large intestine, and increasing susceptibility to macromolecule passage and bacteremia.

## Introduction

The intestinal epithelium and associated immune system have important barrier functions through life, with immunological signaling pathways acting both as a defense against luminal pathogens, or favoring tolerance to food antigens and commensal microorganisms [1]. At birth, the human gut is more permeable than in adult, and immunoincompetent [2], [3]. Bacterial colonization together with endocrine and nutritional factors drive mucosal immune system development, and stimulate growth and renewal of gut epithelium [3]–[5]. Among these factors, glucocorticoids (GC) play important roles for the maturation of digestive and absorptive functions, and stimulate morphogenesis in the small intestine and the colon in human [6]–[8] as well as in rodents [9]–[11]. In rodents, these beneficial activities occur during the first two weeks of life, a period characterized by high responsiveness to GC in various organs including the gut, with low levels of circulating corticosterone (CORT) [12], [13]. In comparison, because the human gut is fully developed at term compared to rodents, a period of GC sensitivity appeared earlier during prenatal development, between the second and third trimester of gestation [7], [8], and postnatal GC administration has trophic effects on the immature gut in preterm infants [6]. From animal studies, a close contact between dams and the litter appears essential for the development of an effective gut barrier for life [14]. Indeed, maternal separation (MS) repeated daily before weaning increased total gut and colonic intestinal epithelial permeability in adulthood, enhancing the risk of intestinal diseases [14]–[17]. In a recent study, CORT injections in adult rats mimic increased gut permeability evoked by chronic MS [18], but whether GC increased gut permeability in neonates, including preterm babies, has not been explored yet. In humans, a mother-infant separation is recognized as a stress factor for the newborn [19], [20]. Nevertheless, a transient MS shortly after birth is of common practice in delivery room for premature babies, after caesarean, as well as in postpartum routines when medical care is required for infant or the mother [19], [21]. Yet, there is no study with stress-based animal models aimed at investigating the consequences of a single MS on the developing gut taking into account the period of postnatal development and endogenous GC sensitivity.

Neonatal rodent models have great potential for mechanistic research on the direct influence of transient MS and associated CORT release on an immature gut barrier, because the hypothalamo-pituitary-adrenal (HPA) axis in early postnatal life is found less responsive to environmental factors compared to the adult, except for maternal separation [22], [23]. Epithelial permeability in the rodent intestine is high at birth as observed in human [24]–[27]. Spontaneous and facilitated bacterial translocation (BT) to mesenteric lymph nodes (MLN) occurs in early life, and peaked at postnatal day (PND) 7, while systemic organs remained sterile [28]. This supports the coordination between epithelial permeability and bacterial colonization for the development of mucosal immune tolerance and pathogen recognition [29]–[31]. One of the aims of the current work was to investigate the immediate impact of a single 4 hours-MS in neonate rats on the epithelial barrier integrity, and whether intestinal CORT responsiveness represents a risk for bacteremia. However, the rat gut at birth is morphologically immature, and resembles that of an early preterm human infant [32], [33]. A rise in CORT plasma level after 14 days of life stimulates intestinal morphogenesis, then reach a functional barrier after three weeks [13], [32]. In the current study, neonate rats at PND10 were used as a model for preterm human newborns, while rat pups at PND20 show comparable intestinal barrier maturity to that observed in full-term human babies during the first week of postnatal life [33]. Specifically, we compared the impact of a single 4 hours-MS session between PND10 and PND20 pups on gut epithelial permeability, and translocation of bacteria to systemic organs. Our study has been performed in relation to basal and MS-induced plasma CORT levels, gut sensitivity to the glucocorticoid receptor (GR) agonist dexamethasone (DEX) using dose-response, and GC receptors expression in the small intestine and the colon.

## Materials and Methods

### Ethic Statement

All experiment protocols were approved by the Local Animal Care and Use Committee of Institut National de la Recherche Agronomique (TOXCOM0035/EH), and conducted in accordance with the European directive 2010/63/UE.

### Animals

Primiparous pregnant female Wistar rats on gestational day 15 were obtained from Janvier (Le Genest St Isle, France). Rats were housed individually with their litters in polypropylene cages at 23°C±1°C on a 12∶12 hour light/dark cycle. Food (UAR Pellets; Epinay, France) and water were available *ad libitum*.

### Single Maternal Separation Procedure

After delivery (PND1), litters were randomly assigned to maternal deprived or sham groups. A single 4 h MS was performed once from 8∶00 to 12∶00 h at PND10 or PND20, during which pups were removed from their dams, and individually housed at 28°C±1°C (single MS group). Sham pups were handled identically, but were maintained with their dams during the 4 h period.

### Experimental Design and Drug Treatment

In a first series of experiments, total intestinal permeability (IP) to fluoro isothiocyanate (FITC)-Dextran 4 kDa (FD4) was measured *in vivo* every 10 days from PND10 to PND50 (5 groups of 7–28 pups at each time point) in male and female offsprings in normal breeding conditions (i.e. left undisturbed); two additional groups of PND10 and PND20 pups were used for qPCR analysis of GR expression in the colon and the ileum (2 groups of 6–8 pups). In a second series of experiments, groups of pups at PND10 or PND20 were assessed for total IP to FD4 at 4, 8, 12 and 24 h after the beginning of MS or sham procedure (n = 4–10 per time point and sex). Two others groups (n = 6–10 per sex) were sacrificed immediately after MS or sham procedure for *ex vivo* determination of colonic and ileal permeability by Ussing chambers. Additional groups were used for determination of plasma CORT concentrations at 1, 2, 4, 8 and 12 h following MS or sham procedure (n = 3–10 per time point). In a third series, *in vivo* IP to FD4 was determined in male and female PND10 pups with or without MS : 4 groups of 7–17 rats per sex were pretreated with ML7 (1 mg/kg body weight (BW) i.p. in 10 µl; Sigma), an inhibitor of myosin light chain kinase (MLCK), or the vehicle (NaCl 0.9%) at 24, 12 and 1 h before *in vivo* permeability measurement; 4 additional groups of 3–7 rats per sex were treated with the GR antagonist RU486 (2 mg/kg BW s.c. in 0.1 ml; Sigma) or vehicle (olive oil) at 12 and 1 h before assessing *in vivo* IP to FD4. In a fourth series of experiments, BT was assessed 24 h after MS or sham procedure in PND10 and PND20 pups (n = 8–14 per group) pretreated with ML7 or its vehicle (0.9% NaCl) the day of MS session. In a fifth series of experiments, a dose-response study to a single injection of dexamethasone (DEX, 0.01 to 2.5 mg/kg BW s.c in 0.1 ml olive oil; Sigma) was performed in PND10 and PND20 male and female pups (n = 3–10 pups per dose) for IP to oral FD4 12 h later.

### *In vivo* Intestinal Permeability

Total IP was measured *in vivo* in rat pups orally given with FITC-Dextran 4 kDa (FD4∶750 mg/kg BW; Sigma) [27]. Four hours later, a sufficient transit time for FD4 recovering in the colon lumen, blood samples (200 µl) were collected from the facial vein using heparin-coated capillaries (SARSTEDT**,** Marnay, France). Mucosal-to-blood passage of FD4 was determined by measuring plasmatic FD4 concentration using an automatic Infinite M200 microplate reader (Tecan, Austria) (Ex 485 nm; Em 525 nm).

### Ussing Chamber Experiments

Colon (distal region) and ileum (2 cm above caecum) segments of 1 cm length were mounted in Ussing chambers (Easymount, Physiologic Instruments, Hamden, USA) as previously described [34]. Epithelial permeability to small and large molecules was measured through the mucosal-to-serosal passage of FD4 and intact Horseradish peroxidase (HRP) 44 kDa (Sigma), respectively, added simultaneously in the mucosal compartment. After 20 min of equilibration, 600 µl of buffer solution on the mucosal side was replaced by 300 µl of FD4 (2.2 mg/ml) and 300 µl of HRP (0.4 mg/ml). Electrical parameters, including potential difference, short-circuit current (Isc) and total electrical resistance (R), were recorded at regular intervals during the 2-hour period of experimentation. Epithelial permeability to FD4 was determined by measuring the fluorescence intensity at 485 nm/525 nm using an automatic Infinite M200 microplate reader (Tecan). Epithelial permeability to intact HRP was determined by an enzymatic assay [35] for specific HRP activity found in the serosal and mucosal compartment with microplate reader (Tecan). Permeability was calculated as the ratio of flux/concentration, as previously described [36], and expressed as cm/second.

### Blood Sampling and Plasma Corticosterone

Blood samples (200 µl) were collected from the facial vein as described above. Plasma was isolated by centrifugation (10 min, 2500×g), and corticosterone (CORT) concentrations were determined by enzyme immunoassay (Immunodiagnostic System, Paris, France) according to the manufacturer’s instructions.

### Bacterial Translocation

Immediately after sacrifice, the liver, spleen and MLN were harvested, weighed and homogenized (Fastprep, Ozyme, France) under sterile conditions. Dilutions were plated onto standard trypcase soy agar and Schaedler agar with 5% sheep blood (Biomérieux, France) for aerobic and anaerobic conditions, respectively, and incubated at 37°C for 48 h. The number of colony forming unit (CFU) was counted, and BT expressed as log CFU per gram of tissue (±SD). The detection limit was 1.63 CFU/g of liver, 1.36 CFU/g of spleen and 2 CFU/g of MLN.

### Real-time qPCR

Total RNA was prepared from ileum (2 cm above the caecum) and distal colon with RNeasy mini kit (Quiagen, Courtaboeuf France). Total RNA was reverse-transcribed using the High Script reverse transcription Supermix (Biorad, Marnes-la-coquette, France). Primer set for GR was: forward 5′TCTGGACTCCATGCATGAGG3’, reverse 5′TCCAAAAATGTCTGGAAGCAGT3’ (annealing temperature 60°C). The qPCR assays were performed with IQ Syber Green Supermix (Biorad) on the CFX96 (Biorad). qPCR data were normalized by TATA-box binding protein (Tbp) expression levels and analyzed using 2^−ΔCt^.

### Statistics

All analyses were done using GraphPad Prism 4 software (GraphPad; San Diego, USA). Intestinal permeability and plasma CORT in response to MS were expressed as the mean±SEM, and data analyzed by ANOVA and Tukey tests for *post hoc* comparisons. BT was expressed as the mean±SD and examined for significance using the chi square test and Fisher exact test. qPCR data were analyzed by Student t-tests. A *P* value <0.05 was considered significant.

## Results

### *In vivo* Intestinal Permeability during Development

**Figure 1:**
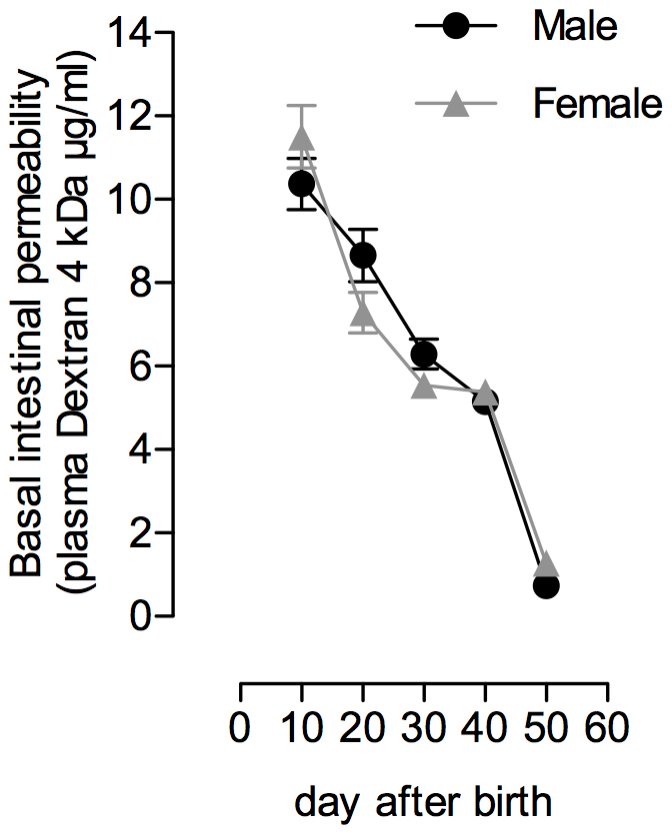
*In vivo* intestinal permeability to oral FD4 during development. Data show the progressive decrease of IP to FITC-Dextran 4 kDa (FD4) every 10 days from the mild-lactation period (postnatal day (PND) 10) to adulthood (PND50). Data are expressed as the mean of plasma FITC-Dextran concentration (µg/ml)±SEM. Numbers of animals per group and sex: PND10 (n = 22–26), PND20 (27–28), PND30 (n = 12–16), PND40 (n = 10–12) and PND50 (n = 7–12).

In basal conditions (without MS), a similar age-related decrease of total IP to oral FD4 was observed in male and female rats from mid-lactation (PND10) to adulthood (PND50) (Figure 1). In PND10 pups, basal IP for FD4 was of 10.4±0.6 and 11.5±0.7 µg/ml in males and females, respectively. From PND10 to PND30, permeability to FD4 progressively decreased (−39% and −52% in male and female, respectively; p<0.001), to display similar levels in both gender at PND40 (5.2±0.3 *vs.* 5.4±0.2 µg/ml in males and females, respectively). From PND40 to PND50, a sharp decrease of IP to FD4 occurred in both sexes, with plasma FD4 concentration at PND50 of 1.3±0.3 µg/ml in females (−76% *vs.* PND40; p<0.001) and 0.7±0.2 µg/ml in males (−87% *vs.* PND40; p<0.0001), thus approximately 10% of PND10 levels.

### Effect of a Single MS on *in vivo* IP and Plasma CORT Levels in PND10 and PND20 Rats

**Figure 2:**
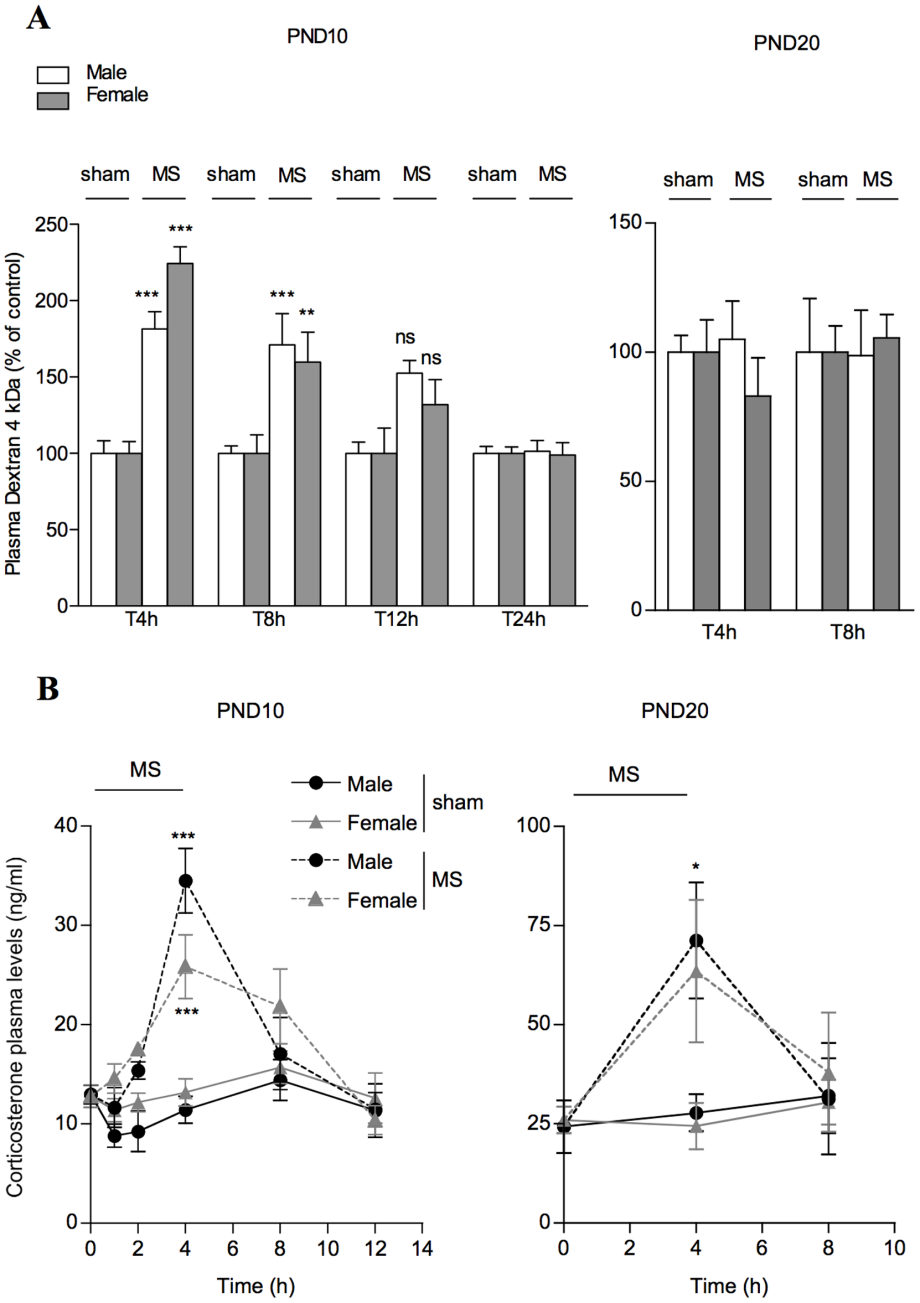
Effect of a single MS for 4 h on IP and plasma CORT in PND10 and PND20 rats. (A) Data show *in vivo* IP to FD4 immediately after MS (T4 h), and after the pups were returned to their dams (T8 h, T12 h and T24 h). Note that only PND10 rats displayed increased IP in response to MS procedure. Data are mean±SEM (7–10 animals per group). **P<0.01 ***P<0.001 compared to corresponding sham controls. (B) Blood samples from MS and sham pups were obtained in rats throughout the MS procedure lasting for 4 h, then every 4 h for 8 to 12 h after the pups were returned to their dams. Note that basal plasma CORT levels in male and female PND10 were lower than in their PND20 counterparts. In both PND10 and PND20 rats, circulating CORT increased in MS rats soon after they were removed from their mother, and peaked at 4 h. Data are expressed as the mean±SEM in 3–10 pups per time-point. **P<0.01***P<0.001 compared to basal.

In PND10 pups, a single MS significantly increased IP to FD4 in male and female rats (+82 and +125%, respectively; p<0.001 *vs.* corresponding controls) immediately after the end of MS procedure (i.e. T4 h) (Figure 2A). This effect persisted 4 h later at T8 h (+71 and +60% in males and females *vs.* controls; p<0.01), and returned to basal values after 12 h. At PND20, a single MS did not change IP to FD4 in both sexes (Figure 2A).

At PND10 under basal conditions (i.e. before MS), male and female pups showed comparable plasma CORT concentrations (13±0.9 *vs.* 12.8±1.1 ng/ml, respectively) (Figure 2B). A single MS progressively increased plasma CORT levels in both sexes, with maximal levels (i.e. 2–3 fold increase compared to sham pups) at the end of MS procedure (Figure 2B). At PND20, higher basal CORT levels were observed in males and females (24.3±6.8 and 26±3.4 ng/ml, respectively) compared to PND10 rats (p<0.01) (Figure 2B), and a 2 fold increase in plasma CORT concentrations was reported 4 h after the beginning of MS compared to PND20 sham pups, then returned to basal values within 4 h after the end of MS procedure (Figure 2B).

### Effect of MS on Colonic and Ileal Permeability in PND10 Rats

**Figure 3:**
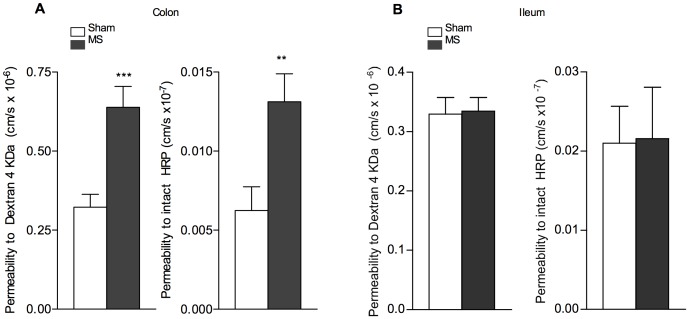
Effect of a single MS on colonic and ileal permeability to FD4 and intact HRP in PND10 rats. Ussing chambers measurements of mucosal-to-serosal permeability to Dextran 4 kDa and HRP 44 kDa in (A) colonic and (B) ileal segments of PND10 pups immediately after MS (T4 h). Note that MS increased FD4 and intact HRP permeability in the colon, but not in the ileum. Pooled data of both genders are shown and are expressed as the mean of permeability to FD4 (cm/s x10^−6^)±SEM in 9–16 animals per group, and HRP (cm/s × 10^−7^)±SEM in 6–17 animals per group. *P<0.05 **P<0.01 compared to corresponding sham controls.

Because the colon is a reservoir of complex microbial composition, and an abundant source of potentially detrimental ligands and antigens for the organism [1], [37], we assessed the effect of MS on specific epithelial permeability to small and large molecules in the colon and the ileum by Ussing chambers (Figure 3). Because no difference between male and female were observed, whatever the intestinal segment, the figure 3 shows data pooled from the two sexes. A single MS induced a 2-fold increase of permeability to colonic Dextran 4 kDa (0.64±0.07 *vs*. 0.32±0.04 cm/s × 10^−6^ compared to sham pups; p<0.001), and to intact HRP 44 kDa (0.013±0.002 *vs*. 0.006±0.001 cm/s×10^−7^, respectively; p<0.01) in PND10 pups (Figure 3A). In contrast, no significant change in epithelial permeability to Dextran 4 kDa or HPR 44 kDa was observed in the ileum of MS pups (Figure 3B).

### Effect of ML7 and RU486 on *in vivo* IP Induced by MS in PND10 Rats

**Figure 4:**
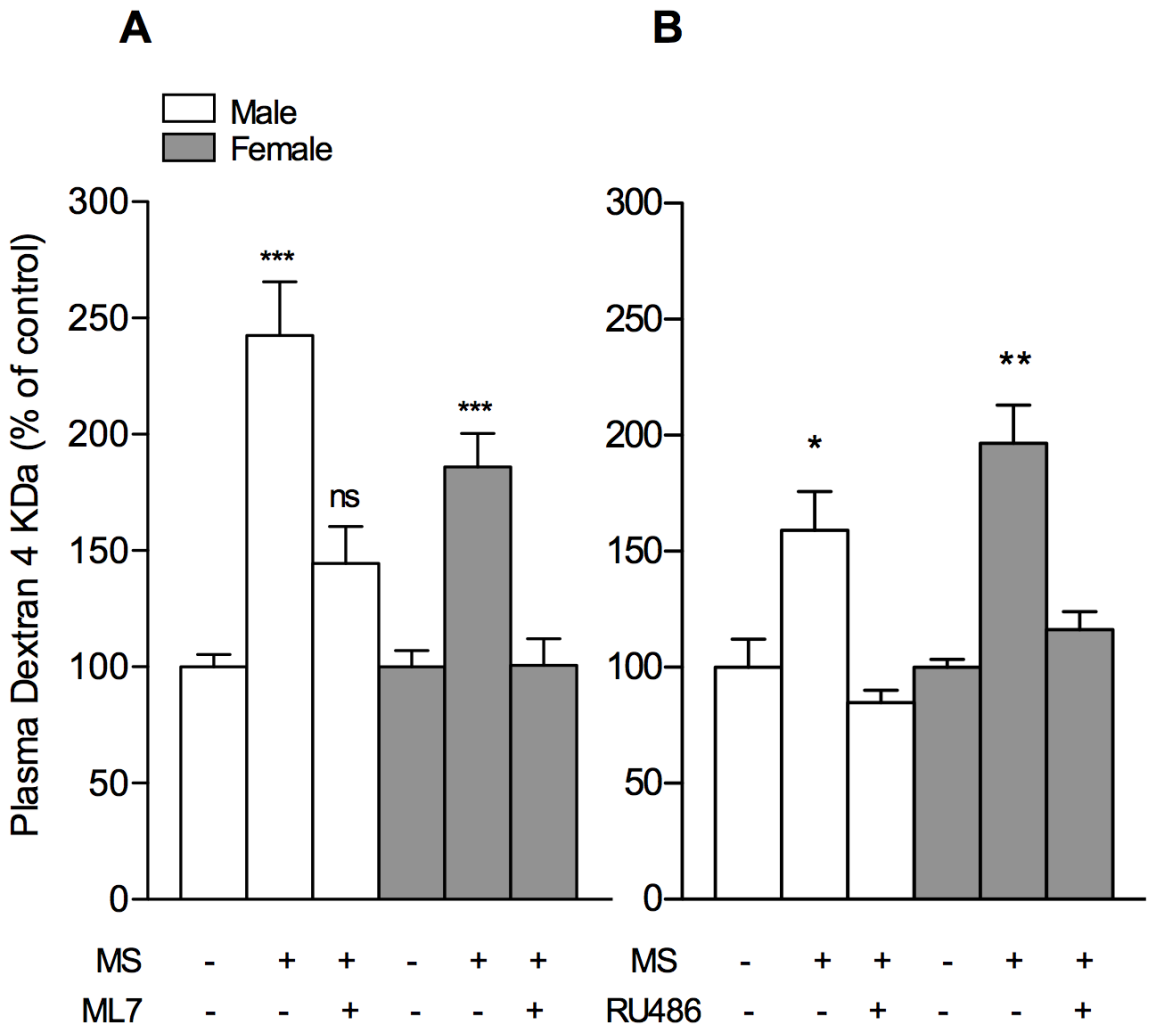
Effects of RU486 and ML7 on MS-induced increase of IP to FD4 in PDN10 rats. Treatment with (A) ML7 (1 mg/kg/d in 0.9% NaCl i.p. at 24, 12 and 1 h before IP measurement), and (B) the GR antagonist RU486 (2 mg/kg/d in olive oil s.c. at 12 and 1 h before IP measurement) prior to MS prevented the IP increase in response to MS. Values are mean±SEM (n = 7–17 and n = 3–7 pups per group for experiment A and B respectively), and *P<0.05 **P<0.01, ***P<0.001 compared to their respective controls.

Previous studies emphasized a pivotal role of MLCK in stress-induced increase of gut permeability in adult rat [38]. In the current study, MS-induced increase of IP to FD4 in PND10 pups was prevented by prior administration of ML7, a specific inhibitor of MLCK (Figure 4A), while the vehicle only (0.9% NaCl) had no effect (not shown). Similarly, RU486 treatment to block GR prior to MS procedure totally prevented the increase of IP to FD4 in response to MS (Figure 4B). The vehicle of RU486 (olive oil) had no effect on MS-induced increase of IP, and neither RU486 nor ML7 treatments altered basal epithelial permeability in PND10 pups in the absence of MS procedure (not shown).

### Intestinal GR Expression and Dose-response Study of DEX on *in vivo* IP in PND10 and PND20 Rats

**Figure 5:**
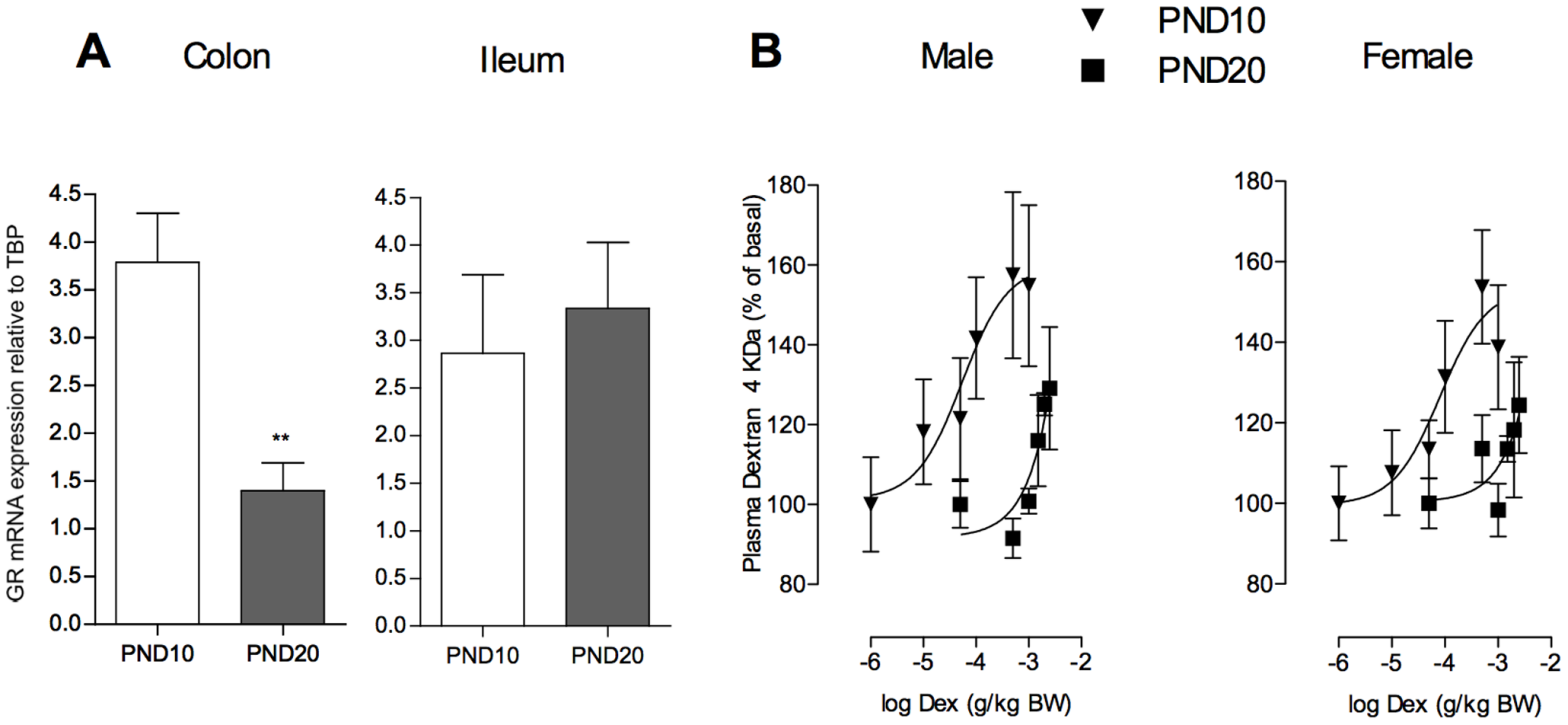
Dose-response study of DEX on IP to FD4 and GR expression in the small intestine and the colon. (A) qPCR results for GR mRNA using total RNA from colon and ileum lysates of PND10 and PND20 female rat pups. (B) Total *in vivo* IP to Dextran 4 kDa (FD4) was measured following subcutaneous injections of Dex (0.01 mg to 2.5 mg (kg BW)^−1^ for 1 day) at PND10 or PND20. In both sexes, note the decreased sensitivity to GC receptor stimulation evoking IP increase to FD4 at PND20 compared to PND10. Data are expressed as the mean±SEM (n = 3–10 pups per group).

A recent study in adult rats reported a region-specific distribution for CORT effects along the GI tract, mainly targeting the colonic epithelium permeability in stress conditions [18]. Compared to PND10 pups, no significant difference in basal expression of GR mRNA was observed in the ileum of PND20 neonates. In contrast, a 70% drop in mRNA levels occurred in the colon from PND10 to PND20 of age (Figure 5A). Changes in IP to FD4 in response to GR stimulation between PND10 and PND20 rats were then studied dose-dependently with the GR agonist DEX. At PND10, DEX significantly increased total IP to FD4 (Figure 5B). Analysis of the sigmoid dose-response curves revealed a median-effective dose (ED50) of 0.1 mg/kg BW in both sexes, and maximal stimulation at 0.5 mg/kg BW. At PND20, we did not observe any increase of IP to FD4 in response to DEX before 1 mg/kg BW (Figure 5B), showing lower efficacy of GR than reported in PND10 pups.

### Effect of MS and ML7 on Bacterial Translocation in PND10 and PND20 Rats

At PND10, a spontaneous BT to MLN occurred in sham pups, with a basal incidence of 25 to 53%, while both liver and spleen were sterile, whatever the gender (Table 1). Twenty-four hours after MS, a significant BT of aerobic and anaerobic bacteria to the liver and spleen occurred (p<0.05), and ML7 pretreatment prevented this effect (Table 1). At PND20, spontaneous BT to MLN significantly decreased or was absent (basal incidence 0 to 13%), and all animal tested were found negative for BT into systemic organs 24 h after MS (Table 1).

**Table 1:** Comparative effects of single MS on bacterial translocation into the MLN, liver and spleen at PND10 and PND20.

		MLN			Liver			Spleen		
		Sham	MS+NaCl	MS+ML7	Sham	MS+NaCl	MS+ML7	Sham	MS+NaCl	MS+ML7
**Aerobic bacteria**										
PND10	male	3/12(2.5)	8/12^ns^(2.3)	2/8(1.1)	0/12	7/12^a^(2.2)	0/8	0/12	7/12^a^(1.9)	0/8
	female	7/13(2.1)	10/14^ns^(3.2)	1/10(3.7)	0/13	9/14^a^(2.0)	1/10(2.1)	0/13	7/14^a^(2.3)	1/10(2.3)
PND20	male	1/8(2)	1/8(2.8)		0/8	0/8		0/8	0/8	
	female	1/8(4.5)	0/8		0/8	0/8		1/8(3.5)	0/8	
**Anaerobic bacteria**										
PND10	male	4/12(1.9)	9/12^ns^(2.5)	3/8(1.3)	0/12	8/12^a^(2)	0/8	0/12	8/12^a^(1.7)	0/8
	female	7/13(1.8)	12/14^ns^(3)	2/10(2.7)	0/13	9/14^a^(1.9)	2/10(1)	0/13	8/14^a^(1.6)	1/10(1.4)
PND20	male	0/8	0/8		0/8	0/8		0/8	0/8	
	female	1/8(5)	0/8		0/8	0/8		1/8(3.4)	0/8	

Expressed as the number of positive organs. Mean number of bacterial colonies indicated between parentheses (logCFU/g of tissue). Values are mean±SD. MLN: mesenteric lymph nodes; PND: postnatal day; **^a^**significantly different (p<0.05), and ns: not significant from sham controls.

## Discussion

Our study shows a vulnerable window in rats during early postnatal life (i.e. 10-days-old) through which a single episode of mother-infant separation evoked an immediate increase of gut permeability to macromolecules in the large intestine, enhancing the passage of viable bacteria to systemic organs. These MS-effects occurred through a MLCK-dependent pathway controlling cytoskeleton contraction in epithelial cells, and were linked to increased CORT plasma levels during the MS period, and downstream activation of GC receptors (GR) found highly expressed in the colon at PND10 compared to later age at PND20. At PND20, neither intestinal permeability nor bacterial translocation was affected after the same MS procedure. A dose-response study to DEX on epithelial permeability assessed *in vivo* showed a shift in intestinal GC sensitivity from PND10 to PND20 that protect the PND20 pups from deleterious MS-induced stress impacts on the gut barrier.

It is well established that neonatal stress evoked by a daily separation from the dam for two weeks (2–6 h per day from PND2 to 14 or 4 to 21) impairs intestinal barrier integrity at the end of the chronic stress procedure, with long-lasting effects through life [14]–[16], [39]. These stress-based animals models have been developed in understanding how repeated traumatic experiences in early life for human may predispose to intestinal diseases in adulthood [14], [15]. However, authors did not investigate the immediate impact on the gut barrier of pups, i.e. at the end of a MS session applied only once as a model for a transient mother-infant separation in early postnatal days. Indeed in many industrialized countries, the medical decision to separate a newborn baby from the mother around the birth is frequent, motivated by the desire to strengthen supervision after a difficult delivery or a caesarean [19], [21], [40]. In most cases, the newborn return to his/her mother after a few hours while maternal deprivation is extended for premature babies in neonatal care units. Because mother-infant closeness and separation are under discussion for infant health [19], [41], the effects on the immature gut barrier in early life have to be documented. This is of importance for example when intestinal barrier disruption in premature babies is considered as contributing to the pathogenesis of necrotizing enterocolitis (NEC), and perinatal stress has been implicated as a risk factor [42], [43], as well as for full-term neonates who develop NEC [44]. Of interest, although the rat gut at birth is morphologically immature compare to full-term human babies [3], [33], it displays during the first two weeks of life a GC sensitivity that resembles that observed in human preterm babies [6], [8], [12], [45]. This makes the 10-days-old rat pups a good model to investigate whether GC sensitivity in an immature gut may shape barrier disturbances for the newborn. At PND20, the intestinal barrier in rodents has developed a fully activated and functional mucosal immunity faced with intestinal microbiota, and looks like that of an healthy full term baby during the first week of life [33].

Because the primary function of gut epithelium is to provide a protective barrier for the organism against adverse luminal factors for life [1], [3], intestinal permeability is commonly used as a marker of epithelial integrity in human newborns [25], [46], [47]. Intestinal permeability is high at birth in rodents as in humans [24]–[27] while the neonatal gut undergoes rapid growth with immunologic changes for complete functional maturity [3], concomitantly to a decrease in intestinal permeability for closure of the epithelial barrier to the external environment [46]–[48]. To illustrate this developmental gut closure, we first used a single oral load of Dextran 4 kDa (herein FD4) in the rat, and measured plasma FD4 concentrations after 4 h to assess *in vivo* IP along the gastrointestinal tract from PND10 to adulthood. We report an age-dependent decrease of IP to FD4 across this period, without difference between male and female rats. This sequence of epithelial barrier maturation appeared similar to that described in mice using similar oral dosage of FD4 [27], although the murine epithelial barrier showed a four-fold abrupt decrease of IP between the second and third week of life, while epithelial barrier in rats continues to mature until PND40 as described herein. The present data have validated the use of *in vivo* IP measurements to FD4 for investigating the impact of a short-time MS on epithelial barrier integrity at different postnatal ages. Hence, rat neonates at PND10, but not at PND20, showed disruption of the epithelial barrier in response to a 4 h-MS and applied once compared to non-deprived pups, with increased IP to FD4 that continued 8 hours after the MS pups returned to their dams, i.e. 12 hours after the beginning of the MS procedure, and returned to basal values by 24 hours. We then investigated whether epithelial permeability was affected in all intestinal segments after MS procedure in PND10 pups. Compared to the ileum, we reported that permeability to FD4 was significantly increased after a single MS in the colon only. In addition, we showed that epithelial passage for macromolecules of higher molecular weight (herein assessed with HRP 44 kDa) was also enhanced in the colon of PND10 MS pups, while ileal segments displayed no change in permeability to intact HRP in response to MS. Interestingly, a colon-specific alteration of epithelial permeability has been recently reported in the gut of adult rats under chronic stress conditions [18]. In their permeability data, these authors demonstrated that increased colonic permeability in stressed rats was limited to small molecules of 400 Da, while the current study in MS pups at PND10 demonstrated enhanced permeability to macromolecular markers up to 44 KDa. This observation is of particular importance for the newborn health in early life, since the colon provides an abundant source of luminal antigens within the range of molecular weights herein assessed, some of them (e.g. food antigens, bacterial toxins, …) may trigger mucosal injury, and a reservoir for systemic infections [1], [2]. Furthermore, it is also important to note that MS effect on intestinal macromolecular permeability was limited to PND10 pups, since increased gut permeability to FD4 (and consequently to upper molecular weight molecules) was not reported later at PND20. This age-dependent relationship probably reflects a rapid maturation process of the rat intestine between 10 and 20 days of life that strengthens the barrier to macromolecules. Because premature infants displayed underdeveloped barrier function in the gut as in the PND10 rats [32], [33], it is suggested that this makes preterm babies in maternal-deprived conditions more susceptible to macromolecules uptake from the gut lumen.

In adult rats under acute stress, it has been reported that altered gut permeability was dependent upon epithelial cytoskeleton contraction through MLCK activation, a mechanism enhancing the passage of macromolecules in the colon [38]. In PND10 rats, we report the same pathway for MS-induced increase of *in vivo* IP to FD4 since ML7, a specific MLCK inhibitor, completely prevented this effect. In MS pups, the immediate consequence of epithelial barrier disruption was an abnormal translocation of viable bacteria to spleen and liver, while these extraintestinal sites remained sterile in control groups, a normal feature in normal breast-fed PND10 rats as demonstrated by Yajima *et al*. [28]. In accordance with these authors, we report spontaneous BT into MLN during the suckling period in non-deprived PND10 rats, a physiological feature that was not affected by MS procedure in our study. Spontaneous BT into MLN in early neonatal life occurs concomitantly to bacterial colonization of the gut, hence participating to the development of immune tolerance to microbiota [49], and it is well accepted that neonatal stress, even acute stressors such as hypoxia, do not change frequency of basal BT into MLN of pups [28], [50]. However, Yajima *et al*. [28] also showed in PND10 rats that a minimal stress factor related to a cannulation gesture to mimic artificial feeding induced significant BT to the liver (>60% of incidence) [28]. At the same age, we show that a non-aggressive stress induced by a single episode of MS for 4 h was sufficient to evoke BT to extraintestinal sites, including spleen, thus demonstrating systemic passage of viable bacteria. In addition, we report that ML7 pretreatment to block MLCK activity in epithelial cells banned bacterial passage to liver and spleen in MS pups, thus establishing a link between MS-induced gut permeability increase, cytoskeleton contraction, and the transepithelial passage of viable bacteria to systemic organs.

It has been shown that chronic neonatal stress interfered with various modulatory systems involved in the gut maturation, through an array of alterations in the brain-gut axis that are influenced by timing of stress procedure [14], [23], [51]. Previous studies emphasized an important role of peripheral GC in the regulation of intestinal growth [10], [11] while the HPA axis in rodents until two weeks of life is characterized by an hyporesponsive period to various stress factors compared to later ages [22], except for maternal deprivation [23]. Consistent with these findings, PND10 pups in our study showed low levels of circulating basal concentrations of CORT in comparison to PND20 rats. A single MS procedure at PND10 significantly enhanced CORT plasma levels 4 hours after the beginning of the MS procedure, and we report that the GR blockade by RU486 completely prevented the MS-induced increase of gut permeability. This first demonstrated that a single 4 h-MS in 10-day-old pups was a sufficient stress event to increase plasma corticosteroids during gut development despite low HPA axis activity, and that the rise in CORT release is responsible for enhanced epithelial permeability across this period. In contrast at PND20, although MS procedure also increased circulating CORT levels, such release was not accompanied by any epithelial permeability changes in the gut. These findings allowed us to investigate whether this lack of MS response on gut permeability at PND20 may be related to difference in intestinal sensitivity to GR stimulation compared to earlier postnatal ages. A dose-related response of *in vivo* FD4 permeability to DEX administration clearly showed the better efficacy of GR stimulation to increase epithelial permeability in PND10 than in PND20 pups. Indeed, DEX treatment at PND20 was only effective in increasing FD4 passage in rats dosed from 1 mg/kg BW while permeability changes at PND10 occurred from 50 µg/kg of DEX with a median-effective dose of 0.1 mg/kg, and maximal stimulation at 0.5 mg/kg, whatever the gender. This difference in GC sensitivity with age is in line with Chen *et al* [11] who observed that 8 and 10-day-old rats were highly sensitive to corticoid-induced morphological changes in the colon, an effect that disappeared after two weeks of life, a time-point corresponding to PND20 pups in our study. Earlier works on ontogeny of intestinal GC responsiveness in rats also reported a decrease in binding activity to GR from the second to third postnatal weeks of life, to reach stable levels until adulthood [12], [13]. These authors also indicated that the small intestine at PND10 displayed high level of GR during the first two weeks of life, despite no response to MS stress in our study in contrast to the colon at the same age. This is in support of a segment-dependent activity of GR along the gastrointestinal tract during gut development, mainly dedicated to enzyme changes in the small intestine for maturation of digestive functions [12], while GR in the colon appeared more participative to epithelial barrier development (our study and [11]). Furthermore, our results provide evidence for a downregulation of mRNA encoding GR in the colon of rat pups between PND10 and PND20, a developmental change not observed in the ileum. This is consistent with recent observations in adult rats indicating a region-specific role for CORT as a mediator for stress-induced permeability changes in the colon, an effect absent in upper intestinal region where GR protein level is 10-fold less expressed compared with colonic tissue [18]. During postnatal life, we propose a similar gradient for GC effect in the immature gut at PND10, and that a decrease in colonic GR expression in PND20 rat pups likely contributes for silencing MS effects on epithelial permeability, and the lower capability of DEX stimulation to affect IP *in vivo*.

In conclusion, this study demonstrated a critical period for mother-infant separation in early neonatal life on intestinal barrier integrity in rats, resulting from exacerbated sensitivity of the immature gut to stress-induced corticosteroid release. These findings highlight that a mother-infant closeness in early life may positively influence the maturational sequence of the intestinal barrier, and that episodes of mother-infant separation in post-partum care, particularly for premature babies, could transiently compromise epithelial integrity, increasing the infant susceptibility to inflammation or sepsis.
